# Crowd Funding for Orphan Drugs: The Case of Baby Pia

**DOI:** 10.3389/fphar.2021.787342

**Published:** 2021-12-14

**Authors:** Wim Pinxten

**Affiliations:** Research Group Healthcare and Ethics, Faculty of Medicine and Life Sciences, Hasselt University, Hasselt, Belgium

**Keywords:** rare diseases, orphan drugs, ethics, crowd funding, SMA, zolgensma

## Abstract

Medical crowdfunding is a relatively new strategy to obtain access to orphan drugs. The case of Baby Pia, a Belgian girl with SMA type 1 for whom in 2018 more than $ 2.1 million was raised to get her treated with Zolgensma^®^, illustrates well the potential power of medical crowdfunding. But apart from the success in raising money, the case is also of particular importance for the ethical issues it brings to the surface as related to justice, equity, power imbalances, responsibility, accountability, indebtedness and privacy.

## Introduction

Rare diseases are life threatening or chronically debilitating diseases with a prevalence that is equal to or lower than five in 10,000 persons. Due to the small number of potential users -often combined with the need for complicated research-the commercial interest in developing drugs to treat rare diseases tends to be low, unless incentives are provided.

In Europe, specific regulations have been issued to encourage the development of “orphan drugs” for the diagnosis, prevention and treatment of rare diseases. (Regulation (EC), 2000; Commission Regulation (EC), 2000). Although these regulations have been successful in stimulating the development of new therapeutic options for patients with a rare disease, they did not resolve all problems. Many patients still face problems in getting access to orphan drugs, as reimbursement may not be arranged and prices are often much higher than what an individual can reasonably be expected to afford. A striking example is Zolgensma^®^, a single dose gene therapy to treat Spinal Muscular Atrophy (SMA), which comes at a price tag of $ 2.1 million. An amount that clearly exceeds the financial capacity of most families, and probably also goes beyond what can be raised through charity in local networks and communities.

Crowdfunding offers new opportunities to extend the reach and power of fundraising. It aims at collecting small amounts from a large group of donors, who jointly enable projects that would otherwise not be financially viable. Given that individual donations are small, the threshold to join in is low. Several platforms facilitate crowdfunding in return for a commission and/or fee, and some are also open to individuals. The largest platform for personal crowdfunding is gofundme.com, which has been used by more than 50 million donors to aggregately donate over 5 billion dollar.

Crowdfunding is increasingly deployed for medical reasons, (Sisler, 2012; Snyder et al., 2016; Berliner and Kenworthy, 2017; Renwick and Mossialos, 2017; Palad and Snyder, 2019; Fong et al., 2020), including purchasing expensive orphan drugs for patients with a rare disease. At the time of writing this paper, gofundme.com revealed 1398 fundraisers when using the search term “Spinal Muscular Atrophy”, and 569 when using the search term “Zolgensma”.

The potential power of medical crowdfunding has been demonstrated by the case of Baby Pia, a Belgian girl with SMA type 1 for whom more than $ 2.1 million was raised to get her treated with Zolgensma^®^. To our knowledge, this is the most successful medical fundraising so far. But apart from the success in raising money, the case is also of particular importance for the ethical issues it brings to the surface. In this case report, the case description will be followed by an exploration of ethical issues involved.

## The Case of Baby Pia

Baby Pia was born in November 2018. The pregnancy had been uncomplicated, and initially there seemed nothing special to worry about. When she was about 3 months old, however, her mother noticed that she didn’t roll on her tummy or tilt her head. And when she held the baby of a friend, she felt a strength in the other child that Pia didn’t have. Following the advice of a friend, she made an appointment with an osteopath to check things out. In the meantime, she also discussed her observations during a routine developmental follow up visit with a pediatrician, where she was reassured that there was nothing really to worry about, as “all babies develop at their own pace”.

When visiting the osteopath a few weeks later, he refers the parents to a neurologist after having done a few tests. At the first consultation, blood is drawn for DNA-analysis, an MRI and EMG scan are scheduled. While the MRI is normal, the EMG appears to be alarming and the potential diagnosis of SMA is brought up, although confirmation by DNA-test still needs to be awaited. Never having heard of this disease, the parents google, get to the Wikipedia page and are confronted with a stated life expectancy of about 18 months.

In a follow up consultation, the neurologist refers the parents to an academic hospital that can provide specialized care and to a pediatric palliative service. The palliative team comes by to discuss mechanical ventilation and end of life issues, which is very disturbing and leaves the parents in despair. Meanwhile, the parents keep struggling to find the best care for Pia. With the help of a patient association for muscular diseases, they find an academic hospital that can provide adequate medical follow up closer to their home. There, treatment with Spinraza®, the first approved treatment for SMA, is initiated immediately. The neurologist also brings up a European clinical trial of Zolgensma®, a single dose gene therapy that is already approved in the United States but not yet in Europe, but states it is unlikely that Pia will be able to enroll in this study.

The treatment with Spinraza^®^ brings new hope, and Pia is making some progress. When getting the news that Pia won’t be able to join the trial of Zolgensma ^®^, however, the parents are left with the uneasy feeling that more health gain might be possible. On Facebook, they have seen impressive results obtained in children with SMA that have been treated with Zolgensma ^®^ at 4 months of age, and also good results in children that were treated later. This makes them eager to look for alternative options to access Zolgensma^®^. They find that there is a Named Patient Program (NPP) that would enable Pia to get Zolgensma^®^ in the United States. While the NPP bypasses the problem that Zolgensma^®^ is still awaiting European approval, the parents now face a second hurdle: the costs of treatment are not reimbursed and thus to be paid out of pocket by the patient. All efforts to lower the bill fail. There is no clinical trial abroad in which Pia can participate. An application for the Belgian “unmet medical need” program is rejected, because the program only reimburses promising innovative treatments if no alternative treatment is available, and for SMA Spinraza^®^ is already being reimbursed. The Ministry of Health does not react to the numerous calls to facilitate. And finally, it appears impossible to negotiate a discount with Novartis. The only option left is to raise $ 2.1 million.

Having nothing to lose, the parents start looking for options to raise funds. They get well-organized from the start: they do not raise money on their own account, but through a newly established non-profit organization that aims to provide adequate support for Pia and others in a similar situation: “TeamPia”. To coordinate action, they bring together friends and people they know who appear to have very useful expertise and connections. Together, they organize several fund raising initiatives (ranging from selling keyholders and wine, over setting up a gofundme-page, to a fundraiser event) and compose a marketing and communication team to disseminate news about Pia in a consistent way. They spread a call to join “TeamPia” through a website and social media, and efforts are made to attract the attention of regional and national media. As a last strategy, they play Euromillions, because you never know…

The approach of TeamPia turns out to be successful: more and more people start to donate. In less than 2 months, about 100.000 euro is raised. Despite being an impressive amount, however, this only accounts for 5% of the bill. Additional ways to get people donating are being explored, among which donation via text-messages (a common practice in bigger fundraising events organized by national media). They get in touch with the only company that facilitates this technology in Belgium, and learn that they can make use of the services, be it at a service cost of about 30 percent of the amount raised. As the text messages are only considered to be an add-on on the other ongoing fundraising strategies and TeamPia has a poor position in the negotiation, these conditions are accepted. From now on, people can donate 2 euro to TeamPia by sending a text message with “Pia” to dedicated number 4666. In reply, they get a text message stating “Welcome to TeamPia”.

Soon, the texting strategy appears to be promising: during the first weekend, about 20.000 text messages come in. TeamPia keeps on exploring how to get bigger exposure and attempts through indirect network contacts to convince celebrities and influencers to share the call to join TeamPia by sending a text message and donating 2 euro. When world renown DJs, football internationals, singers and many others start sharing the message, the number of incoming text messages increases exponentially within hours. In response, national media starts covering the story and provides the public at large updates on how much money has been raised so far. An ever bigger crowd joins TeamPia. When people inform on social media whether their donation goes entirely to TeamPia, the high commission of telecom providers raises public indignation. In response, all providers agree to skip their commission, which brings TeamPia another step closer to the goal. In the meantime, the number of text messages keeps on booming, and 2 days later 930.000 text messages have come in and sufficient money had been raised to pay for Zolgensma^®^.

In October 2019, Pia got an injection with Zolgensma^®^, and tolerates the treatment well. In the following months, she reaches new milestones: she starts rolling, sitting, talking. She increases the stability of her head, improves motor control in the arms, and also makes some progress in the legs. Today, Pia goes to school and the prefix “baby” no longer holds.

One element that has not been highlighted in our account of Baby Pia’s story so far, is newborn screening for SMA. When Pia was born, newborn screening for SMA was not available in Flanders, the region where Pia lives. If it would have been, the diagnosis would have come sooner, Pia might have had better chances to join the European trial of Zolgensma^®^, and the treatment (with Spinraza^®^) might have been initiated sooner with better results. To date, TeamPia advocates to adopt SMA in the newborn screening.

## Ethical Issues

There is more to the story of Pia than successful fundraising. The case is also a story of how patients who do not suffer from common health problems often struggle for care, also in countries with extensive public health insurance. A story of power imbalances and communication difficulties between patients and governments and pharmaceutical companies. And of the hidden cost of crowdfunding. As such, the case brings several important ethical issues to the surface.

### Justice, Equity and Power imbalances

Facilitating access to an expensive drug for one child while not being accessible for others in the same condition creates obvious inequalities. However, it is one thing to argue that justice requires that all children with a similar health condition and comparable medical needs in the same public health system have the right to the same treatment, it is quite another to argue that it would be unjust for parents to pursue the best possible care for their children. Doing so does not equal being insensitive to the needs of others or taking away resources or opportunities from others. The crowdfunding for baby Pia did not directly drain resources away from other children in need, as it didn’t interfere with public healthcare funding at all. If it did so indirectly -by encouraging people to donate for this action and hereby potentially drafting attention away from fundraisers for others in need-, this reflects a dynamic that is inherent to all charity.

As a Named Patient Program was already existing, the drug was open to European users, and the parents did not bypass the European approval process in an unjustified way.

Arguments that the impressive amount of money could be used in a different way so that more people get help or more health gain is obtained may be relevant to public health insurance, but cannot be translated to private initiatives in which people are free to use their private financial resources for the ends they personally prefer. Individual patients who receive donations as a form of charitable support to their personal needs, need not to have a responsibility in allocating budgets fairly. Moreover, the idea of allocating a budget is not applicable to most medical crowdfunding, as most people are not looking for a budget to spend according to their preferences, but seek to overcome a lacuna in public health insurance. For them, money is just the vehicle to their ultimate end: getting a treatment.

Another justice-related issue are power imbalances. Patients who cannot afford a treatment without the help of a crowd are in a vulnerable position. Their calls for help can remain unanswered as they can easily be ignored by policy and industry. They have a poor position in negotiating with pharmaceutical companies, the government, and fundraising platforms. They don’t have the power to speed up processes, while the regular pace of policy might be too slow to bring timely relief. These power imbalances also make that patients are very uncertain about the effectiveness of their efforts. They have to take the risk of not getting anything useful back for the time and effort they invest in trying to get better care.

### Responsibility, Accountability, Indebtedness

Shared decision making has become a creed of contemporary medical practice. When pursuing new treatments that are not yet approved and/or reimbursed, however, patients tend to fall back on their own. There is hardly any support in finding out what you can do and how you can get organized. Furthermore, essential decisions are no longer backed by policy makers (who have already decided for you that your treatment is offered as a standard treatment) or by a medical team (that considers such standard treatment to be medically indicated). This attributes a bigger responsibility to patients than would be the case for a reimbursed standard treatment.

Next to responsibility, medical crowdfunding also brings in accountability. Accountability for not misleading potential donors, while the only way to reach them is by mobilizing media that has more interests in clear headlines and public indignation than in scientific nuance. Media that strongly edit stories to make them fit their purpose, and hereby sometimes violate reality. Telling your story in all complexity and honesty does not preclude this from happening, and it may not be practically feasible to correct all misinformation. In addition, patients or their parents are held accountable for how donations are being used. What if insufficient amounts are being raised and the treatment remains out of reach (which is not unlikely at all for a treatment as expensive as Zolgensma^®^)? How is the money to be spend there is still left after costs have been paid?

Finally, getting $2.1 from the crowd is not likely to end up in a one way story, as patients or their parents can feel indebted to the donors. This kind of reciprocity is less present in anonymous reimbursement by public health insurance, and can have a significant impact on the lives of the beneficiaries. How do you deal with potential disappointment about the outcome? How do you deal with the persistent feeling of having to do something back, by communicating updates, giving interviews and lectures, and helping others who face the same troubles?

### Privacy

Another important downside of crowdfunding is the loss of privacy. (Palad and Snyder, 2019). To mobilize the crowd, you need to share your story, often up to a level that strongly invades with privacy. It entails the sharing of information about the life and health status of a minor who cannot consent to this. In addition, it requires the investment of a significant amount of time in communicating with the media and the public at large, often at inconvenient times and in the middle of health and family crises. At the same time, you are exposed to the comments of complete strangers, some of which feel free to share rude comments and insults. Such comments, however small their share among the many positive notes, can be impactful and even violent.

## Discussion

Medical crowdfunding is a relatively new strategy to obtain access to orphan drugs. Despite its potential for being successful in collecting the required amounts of money, it must be emphasized that medical crowdfunding also comes with a considerable hidden cost, related to justice, equity, power imbalances, responsibility, accountability, indebtedness and privacy as well as practical issues such as time and effort. Also other ethical issues have been described elsewhere. (Snyder, 2016; Snyder et al., 2016; Snyder et al., 2017; Kubheka, 2020). Finally, crowdfunding makes patients heavily dependent on skills, networks, public appeal and even luck. This also reflects in the case of Baby Pia. While it is impossible to reliably explain for the success, there are a few factors that are likely to have played an important role. The story has a strong appeal, concerning a baby with an incurable condition, a limited life expectancy in absence of treatment, and an existing but unaffordable drug. The parents and their friends were believers, who did everything in their power to give her a future as open as could be. TeamPia was driven by a carefully composed team with essential expertise and access to important contacts. It had a well thought out communication strategy, which truly mobilized the crowd, and not just addressed a local community. By joining TeamPia, also people who were completely disconnected from the family or community could sign up for something big, which allowed people to be proud for being committed to the common goal of beating injustice and making the seemingly impossible happen. And finally, probably also luck played a role: there were for example no important events that would compete for media attention, and the story was picked up by important influencers.

The case of Baby Pia does not reveal a magic formula for getting orphan drugs funded. In absence of such formula, however, there is no equity in chances to benefit from medical crowd funding. By contrast, medical crowd funding entails a high risk of being unsuccessful, while inducing a considerable moral and practical burden.

To be more fair, the access to orphan drugs should not be dependent on the success of crowdfunding. While we may see no fundamental objections to parents striving for the best for their children, as a society we need to approach the problem of expensive orphan drugs differently. We need to secure solidarity, also in absence of the empathy of the crowd, so that all people alike get similar chances. Public health insurance is the platform where this can best be achieved. We need to address power imbalances, preferably with joint forces crossing national borders. And finally, we need to better consider time, as waiting for diagnosis, approval and reimbursement should not take longer than needed.

## Data Availability

The original contributions presented in the study are included in the article/Supplementary material, further inquiries can be directed to the corresponding author.
